# Supplementary material for: Towards unified AI-driven fracture mechanics: the extended deep energy method (XDEM)
Source: Nat Commun. 2026 Aug 15;17:8492. doi: 10.1038/s41467-026-76748-1 (PMC13484471; doi:10.1038/s41467-026-76748-1)
Supplement: Supplementary file 3 [file 41467_2026_76748_MOESM3_ESM.zip › pic/Results/Stress_intensity_factor/Mode2/nn_architecture_comparison_tau_100.pdf]

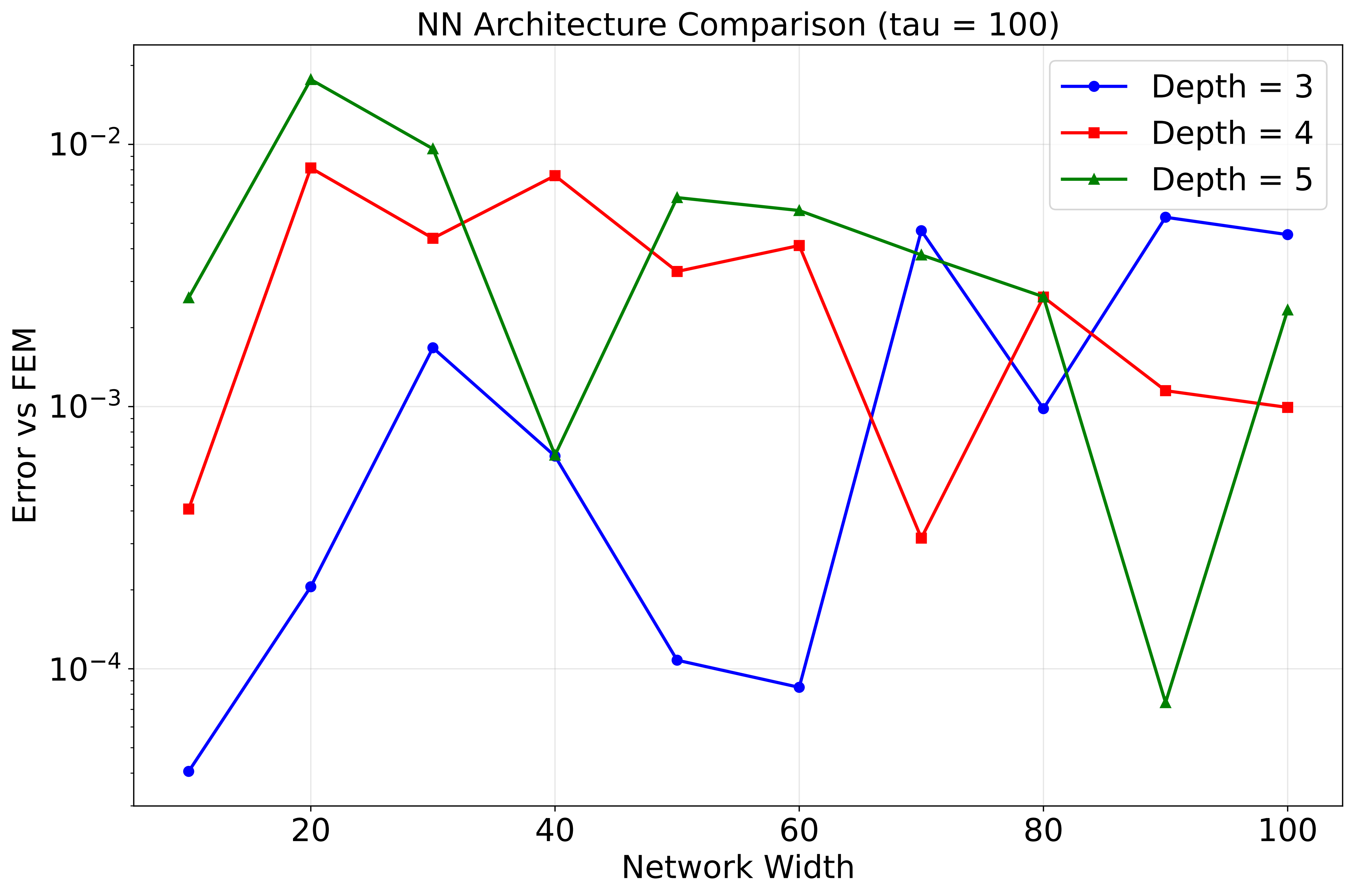
NN Architecture Comparison (tau = 100)
Depth = 3
Depth = 4
Depth = 5
Error vs FEM
10^{-2}
10^{-3}
10^{-4}
20
40
60
80
100
Network Width
